# Generative adversarial network for glioblastoma ensures morphologic variations and improves diagnostic model for isocitrate dehydrogenase mutant type

**DOI:** 10.1038/s41598-021-89477-w

**Published:** 2021-05-10

**Authors:** Ji Eun Park, Dain Eun, Ho Sung Kim, Da Hyun Lee, Ryoung Woo Jang, Namkug Kim

**Affiliations:** 1 Department of Radiology and Research Institute of Radiology, University of Ulsan College of Medicine, Asan Medical Center, 43 Olympic-ro 88, Songpa-Gu, Seoul, 05505 Korea; 2 Department of Convergence Medicine, Asan Medical Institute of Convergence Science and Technology, Asan Medical Center, Seoul, 05505 Korea; 3 School of Medicine, Kyunghee University, Seoul, 02447 Korea

**Keywords:** Information technology, Diagnostic markers, Cancer imaging, Molecular medicine

## Abstract

Generative adversarial network (GAN) creates synthetic images to increase data quantity, but whether GAN ensures meaningful morphologic variations is still unknown. We investigated whether GAN-based synthetic images provide sufficient morphologic variations to improve molecular-based prediction, as a rare disease of isocitrate dehydrogenase (IDH)-mutant glioblastomas. GAN was initially trained on 500 normal brains and 110 IDH-mutant high-grade astocytomas, and paired contrast-enhanced T1-weighted and FLAIR MRI data were generated. Diagnostic models were developed from real IDH-wild type (n = 80) with real IDH-mutant glioblastomas (n = 38), or with synthetic IDH-mutant glioblastomas, or augmented by adding both real and synthetic IDH-mutant glioblastomas. Turing tests showed synthetic data showed reality (classification rate of 55%). Both the real and synthetic data showed that a more frontal or insular location (odds ratio [OR] 1.34 vs. 1.52; *P* = 0.04) and distinct non-enhancing tumor margins (OR 2.68 vs. 3.88; *P* < 0.001), which become significant predictors of IDH-mutation. In an independent validation set, diagnostic accuracy was higher for the augmented model (90.9% [40/44] and 93.2% [41/44] for each reader, respectively) than for the real model (84.1% [37/44] and 86.4% [38/44] for each reader, respectively). The GAN-based synthetic images yield morphologically variable, realistic-seeming IDH-mutant glioblastomas. GAN will be useful to create a realistic training set in terms of morphologic variations and quality, thereby improving diagnostic performance in a clinical model.

## Introduction

Isocitrate dehydrogenase (IDH) mutation status of gliomas is a very important prognostic, diagnostic, and therapeutic biomarker^1^. Although the frequency of IDH mutation in primary glioblastoma is low (~ 8%)^1,2^, noninvasive imaging-based determination of IDH mutation status can predict response to anti-IDH treatment or vaccination^3–6^. In addition, radiologic suspicion of IDH-wild type may predict prognosis in patients with inoperable tumors^5^. Magnetic resonance imaging (MRI) has been shown to distinguish between tumors with wild-type and mutant IDH, but these studies have focused primarily on grade II/III gliomas^7–10^ or included a very limited number of IDH-mutant glioblastomas^11,12^ for visual analysis or deep learning. A multicenter cohort study of 496 patients with glioblastoma showed IDH mutation in 31 (6.3%)^11^, limiting the ability of MRI to train a network to reliably predict IDH mutation status. In consequence, most studies seeking to improve the noninvasive identification of this subtype have lacked sufficient statistical power.

Data augmentation is a key element of deep learning models, and the application of geographic modifications, including rotations, translations, shearing, zooming, and flipping^13^ is designed to deal with unbalanced classes and improve the accuracy of predictions^14^. A generative adversarial network (GAN) is different from conventional approaches that can generate plausible new images from unlabeled original images^15^. GAN learns data distribution from training samples and can generate realistic imaging data that are similar in distribution, but nevertheless differ from the original data; this may constitute an attractive solution of overfitting for small datasets^13,14^. GAN has been applied for reconstructing multi-contrast MR images^16–18^, reducing noise^19^, detecting^20,21^, and tumor grading^22^, but assessment of the morphologic characteristics of GAN-based synthetic data and their ability to classify molecular subtype in a diagnostic models have not been tested. If GAN-generated imaging data reflect the morphologic characteristics of glioblastomas with mutant IDH, while varying in morphologic distribution, then these GAN-generated data can be used for training on future deep learning tasks. The presence of morphologic variations is also indicative of avoiding mode collapse or memorization from GAN algorithms^23^, which would extract meaningful morphologic characteristics and enhance prediction of molecular subtype. To determine whether GAN-produced images reflect the morphologic characteristics of actual tumors, enabling their use as a future training set, a diagnostic model was created from the morphologic characteristics of actual and synthetic data. This model was used to determine whether the synthetic images affect performance and could be validated in an independent dataset. The purpose of this study was to investigate whether GAN-based generated IDH-mutant glioblastomas provide morphologic variations and improve molecular prediction of the IDH status of glioblastomas.

## Materials and methods

This study is reported in accordance with the Standards for Reporting of Diagnostic Accuracy Studies (STARD) 2015 guidelines^24^. The study protocol was approved by the institutional review board of Asan Medical Center, a tertiary referral hospital, which waived the requirement for informed consent because of the retrospective nature of the study.

### Study population

**Figure 1:**
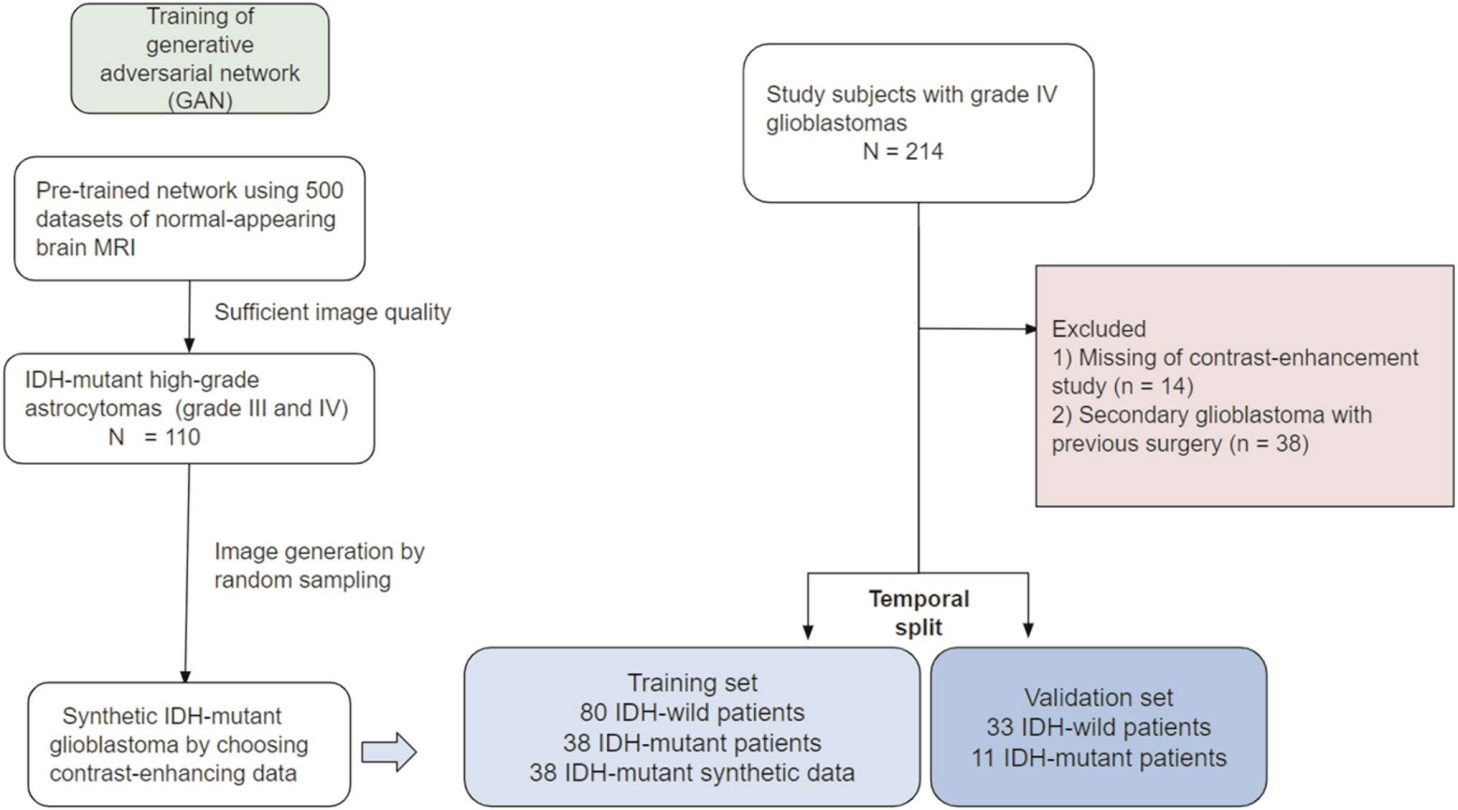
Process for inclusion of the study population and the training dataset for the generative adversarial network.

The study population consisted of a cohort of consecutive patients with histopathologically confirmed glioblastoma who underwent brain MRI from May 2017 to May 2020 (Fig. 1). Patients were included if they were histopathologically diagnosed with glioblastoma and their IDH mutation status was known, according to WHO 2016 criteria^1^. A total of 214 patients met the inclusion criteria. Patients were excluded if (a) pre-operative contrast-enhanced T1-weighted imaging or fluid-attenuated inversion recovery imaging was not performed (n = 14), or (b) they had history of previous surgery (n = 38). The study population consisted of 162 patients, 65 men and 97 women, of mean ± standard deviation age (SD) 56 ± 10.7 years, with 118 patients who underwent brain MRI from May 2017 to January 2019 assigned to the training set, and 44 patients who underwent brain MRI from February 2019 to May 2020 assigned to the validation set.

Patients with IDH-wild type were significantly older than patients with IDH-mutant type glioblastoma, both in the training set (median [interquartile range], 60 [53–64] vs. 47 [38–53] years; *P* < 0.001) and in the validation set (58 [49–64] vs. 42 [31–53] years; *P* = 0.003).

### IDH mutation status

IDH mutation status was analyzed by members of the pathology division of our hospital who were blinded to the radiologic results. The reference standard consisted of immunohistochemical determination of IDH1 (R132H) protein expression^25^. Mutations in the *IDH1* and *IDH2* genes were determined by DNA pyrosequencing at diagnosis^25^.

All patients were tested for 1p/19q co-deletion status and found the 1p/19q co-deletion was negative, indicating astrocytomas.

### Imaging data acquisition

All enrolled patients underwent MRI on a 3.0 T unit (Achieva or Ingenia, Philips Medical Systems) using a 16-channel or 32-channel head coil. The MRI protocols included T2-weighted imaging, fluid-attenuated inversion recovery (FLAIR) imaging, T1-weighted imaging, and contrast-enhanced T1-weighted imaging. The parameters for the T2-weighted imaging are as follows: repetition time (TR)/echo time (TE), 9000/135 ms; field of view (FOV), 240 mm; matrix, 256 × 256; and slice thickness, 4 mm. The contrast-enhanced T1-weighted (CE-T1w) images were obtained at a high-resolution three-dimensional (3D) volume, using a gradient-echo T1-weighted sequence with the following parameters: repetition time (TR)/echo time (TE), 9.8/4.6 ms; flip angle, 10°; field of view (FOV), 256 mm; matrix, 512 × 512; and slice thickness, 1 mm with no gap. The parameters for FLAIR imaging included TR/TE, 9000/135 ms; flip angle, 90°; FOV, 240 mm; matrix, 512 × 512; and slice thickness, 4 mm with no gap.

### Image preprocessing

To prepare the training data, both CE-T1w and FLAIR images were subjected to skull stripping using HD-BET algorithms^26^. Each FLAIR image was co-registered to the corresponding CE-T1w image by within-subject registration using a rigid-body model, image reslicing, and SPM12 software^27^. The CE-T1w, FLAIR, and null images were combined into a three-channel image. A total of 19,595 three-channel images from 110 IDH-mutant patients were fed into the style-based GAN architecture (StyleGAN2) network to simultaneously generate synthetic IDH-mutant CE-T1w and FLAIR images.

### Theory

GANs have been shown to generate realistic images from latent vectors. Although the latent vector sampled from a uniform distribution is traditionally provided to the GAN generator network^28,29^, this approach leads to an unavoidable feature entanglement. Because feature disentangling is required for smooth image generation, StyleGAN first introduced the mapping network, $f: \mathcal{Z}\to \mathcal{W}$, which transforms latent $z\in \mathcal{Z}$ from a uniform distribution to the intermediate latent vector $w\in \mathcal{W}$. StyleGAN also successfully introduced adaptive instance normalization (AdaIN) to the generator network, enabling the computation of the invariant style $y$ from the intermediate latent vector $w$.

Following the success of StyleGAN, StyleGAN2 further improved image-generation quality by redesigning the generator architecture, reducing the common artifacts observed in StyleGAN-generated images. The performance of the StyleGAN2 synthesis network $g$ was improved by introducing several modifications (Supplementary Fig. 1). The applications of bias, noise, and normalization to the constant input at the beginning of the network architecture were removed. Then, bias and noise operations were added outside the styleblock. The AdaIN operation was divided into modulation and demodulation operations. The modulation operation scaled each input feature map of the convolution by its scaling value, which was determined by the incoming style. The demodulation operation normalized each output feature map to the L2 norm of each output channel. With these modifications, StyleGAN2 successfully removed common artifacts that were commonly observed in StyleGAN^30^.

### Contrast-enhanced T1-weighted and FLAIR cogeneration and StyleGAN2 implementation details

Although the generation of multi-modality images is considered favorable, most medical image synthesis studies have focused only on the generation of single-modality images^20,21,23,31^. By combining CE-T1w and FLAIR images into multichannel images, StyleGAN2 generated CE-T1w and FLAIR images simultaneously (Supplementary Fig. 2).

The sizes of the input latent vector $z$ and the intermediate latent vector $w$ were each set at 1 × 512. The output image size was 3 × 256 × 256; the first channel was the CE-T1w image, the second channel was the FLAIR image, and the last channel was the null image. The mapping network consisted of eight fully connected layers. Leaky ReLU activation with alpha = 0.2 was used for activation function and bilinear filtering for all up and down sampling layers. The learning rate was set at $2 \times {10}^{-3}$. An Adam optimizer was used with hyperparameters ${\beta }_{1}=0, {\beta }_{2}=0.99, \varepsilon = {10}^{-8}$ and minibatch size 32. Since there is no golden rule for evaluating image quality, we optimized the hyperparameters following two methods: First, Fréchet inception distance (FID) score was measured, which are designed for the image quality assessment of synthetic images^32^. The FID score calculates discrepancy of the two distributions in the high dimensional feature space of the pretrained Inception V3 classifier. The lower FID score means higher similarity between two distributions. The FID score smoothly decreased from 327 points to below 9.5 points as the network was iteratively trained. The FID score loss is shown in the Supplementary Fig. 3. Second, visual Turing tests were performed for synthetic images by two expert neuroradiologists, aimed to less than 60% for synthetic images. At each session, 50 images of samples were chosen from synthetic images for image quality assessment. At the fifth evaluation session, Turing tests of the imaging data showed that the correct classification rates by readers 1 and 2 were 55% and 62%, respectively.

The network was trained on a NVIDIA TITAN RTX 24 GB GPU. The training of 80,000 images took approximately 25 min, and the generation of 100 synthetic images took approximately 8 s. The network was iteratively trained for 4 million images. The code was modified from the original paper^30^, which is available at https://github.com/NVlabs/stylegan2. All experiments were implemented with the official tensorflow code of StyleGAN2 provided by the NVIDIA Corporation.

### Sample size and rationale for the training network

StyleGAN2 was initially developed to train data using 500 datasets of normal appearing brain MRI, obtained from 393 men and 107 women of mean ± SD age 49.4 ± 12.1 years. These datasets included contrast-enhanced T1-weighted and FLAIR images that were obtained for evaluation of brain metastases in patients with lung cancer, with all patients diagnosed as negative for metastases in brain parenchyma. The images created from StyleGAN2 were reviewed by two experts (J.E.P. and H.S.K., with 5 and 20 years of experience, respectively, in neuro-oncologic imaging). These evaluations confirmed that the generated imaging data yielded realistic images without artifacts.

The sample size was set at 100 for the training network to provide realistic data. Thus, synthetic data for IDH-mutant glioblastomas were generated from a dataset consisting of images of 110 patients, 57 men and 53 women, of mean ± SD age 54 ± 12.3 years, with WHO grades III and IV IDH-mutant high-grade astrocytomas, including 49 IDH-mutant glioblastomas. The synthetic imaging data reflected the morphologic features of IDH-mutant type astrocytomas, as shown in Supplementary Fig. 4.

### Imaging analysis

Training was continued until the two expert radiologists found it difficult to distinguish between real and synthetic data.

#### Evaluation of reality

Turing tests of each dataset were performed independently by the two observers 2 weeks before morphologic assessment. The evaluation was binary, with a score of 0 indicating that the data appeared fake and seemed to consist of GAN-generated synthetic data, whereas a score of 1 indicated that the data appeared real^33^. The correct classification rate and misclassification rates were calculated.

#### Morphologic assessment

**Figure 2:**
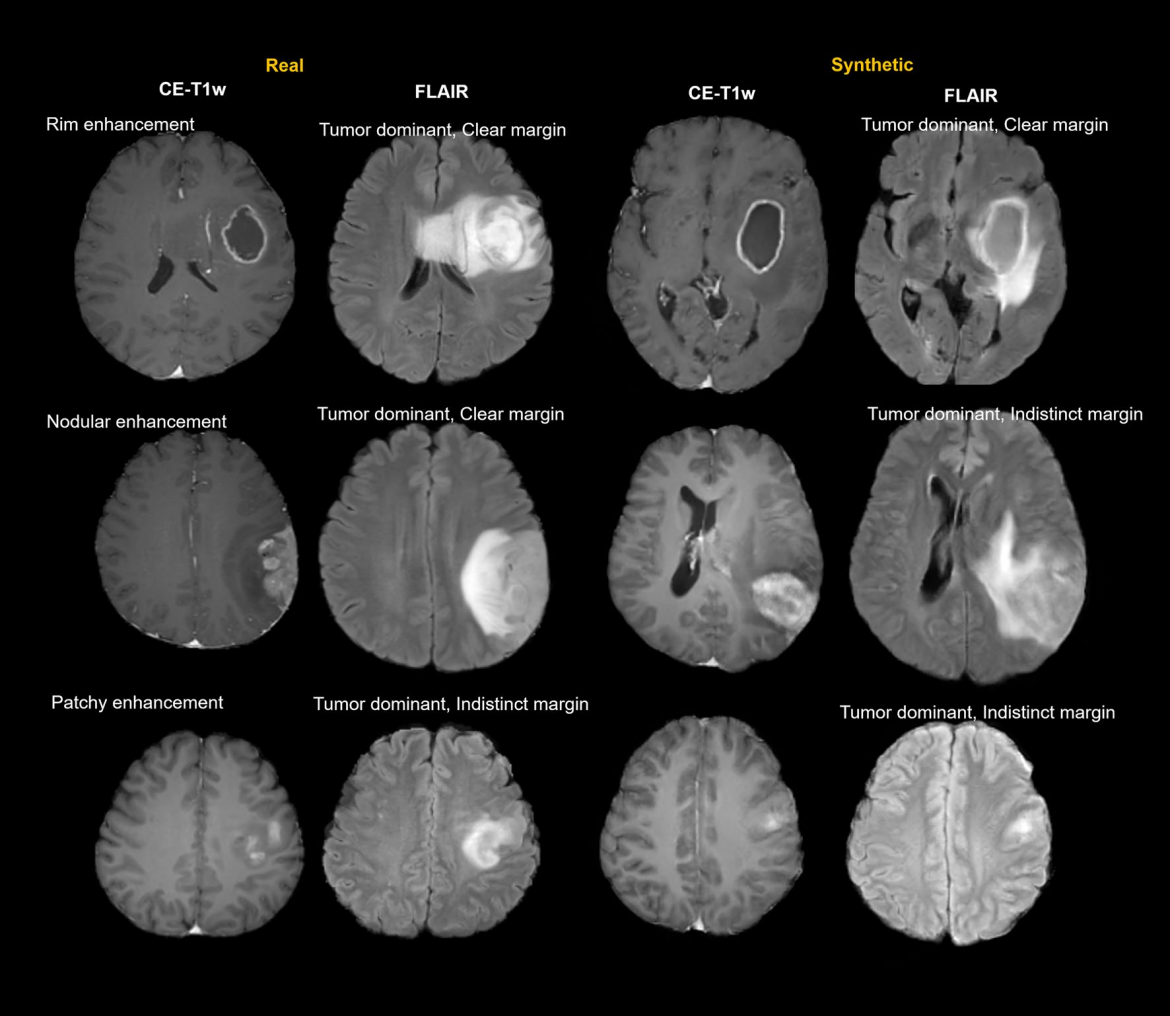
Morphologic characteristics of real IDH-mutant glioblastomas (left) and synthetic IDH-mutant glioblastomas generated by a generative adversarial network (right) based on contrast-enhanced T1-weighted (CE-T1w) and paired FLAIR images. (**A**) CE-T1w images showing different contrast patterns of rim enhancement, thick nodular enhancement, and patch enhancement. (**B**) FLAIR images showing types of surrounding high signal intensity (tumor dominant and edema dominant) and margins of non-enhancing lesions (clear and indistinct). Although the appearances of synthetic images are similar to those of real images, there were no exact matches.

A radiologist (H.S.K., with 22 years of experience in neuroradiology) who did not participate in any other image review in this study selected single 2D FLAIR-weighted and contrast-enhanced T1-weighted images to be reviewed, with real and synthetic imaging data randomly shuffled. Two observers (J.E.P. and D.L., with 5 and 1 years of experience, respectively, as board-certified neuroradiologists) independently reviewed 200 MRI datasets, while being blinded to diagnosis and the evaluations of other observers. Feature categories were adapted from previous studies of IDH mutations in WHO grade II/III gliomas^8–10^. Tumor location was specified by epicenter, with locations grouped according to the frequency of IDH mutation, thereby reducing the number of variables for statistical analysis. The locations included the frontal or insular cortex, the thalamus or brainstem, and others. Patterns of contrast enhancement included rim enhancement surrounding central necrosis, nodular enhancement, and partial patchy enhancement. The areas surrounding regions of high signal intensity on non-enhancing FLAIR images were recorded as tumor dominant or edema dominant, and the margins surrounding these regions as clear or indistinct. Representative cases generated from synthetic data are shown in Fig. 2.

### Statistical analysis

#### Distribution of morphologic features

We tested the distribution of data using the Shapiro–Wilk test. Because the data rejected normality, we recalculated the comparison of demographic and imaging features using non-parametric methods with the Mann–Whitney U test for continuous variables and chi-square test for categorical variables. The data are expressed as the count and median with interquartile range. All statistical analyses were performed using R software (version 3.6.1), with *P*-values < 0.05 regarded as statistically significant.

#### Significant predictors for IDH mutation

Inter-observer agreement on morphologic categories was evaluated by Cohen κ testing. Values of < 0 indicated no agreement, whereas values of 0–0.20, 0.21–0.40, 0.41–0.60, 0.61–0.80, and 0.81–1.0 indicated slight, fair, moderate, substantial, and almost perfect agreement, respectively. Morphologic categories with κ values ≥ 0.5 were subject to univariable analysis. Discordant morphologic categories were subsequently resolved by consensus for variables in the model.

Univariate logistic regression analyses were performed to test whether morphologic criteria could predict IDH mutation status. Nagelkerke (Pseudo) *R*^2^ was used as a summary statistic to determine the degree to which the overall model predicted the variation in IDH mutation positivity. Parameters significant in univariable analysis, defined as those with *P* < 0.05, were subsequently entered into the multivariable analysis. Multivariable binomial logistic regression was performed to predict IDH-mutant vs. IDH-wild type glioblastoma using a stepwise elimination process. Models were built separately for real IDH-wild type and IDH-mutant data (n = 118, model 1), real IDH-wild type and synthetic IDH-mutant data (n = 118, model 2), and real IDH-wild type, real IDH-mutant, and synthetic IDH-mutant data (n = 156, model 3).

#### Diagnostic performance for IDH mutation

Using the results from the multivariable regression analysis for each model, the probability of IDH mutation positive status was calculated for individual patients in the validation set. The diagnostic performance of the multivariable model was determined by calculating the area under the receiver operating characteristics (ROC) curve, with the diagnostic threshold determined using the Youden index. The three above models were compared.

Additionally, univariate logistic regression analysis was performed to determine whether age could predict IDH mutation status. The age-based prediction was subsequently combined with the image-based prediction using a logistic regression classifier in the training set with real data (model 1) and in the validation set.

## Results

### Patient demographics

This study included 162 patients, consisting of 65 men and 97 women. Of these, 118 patients were included in the training set and 44 patients in the validation set. Table 1 shows the demographic characteristics of these patients, as well as the imaging characteristics of the real and synthetic datasets. A video (Online Supplement) shows continuous synthetic tumor on contrast-enhancing T1-weighted and FLAIR images.

**Table 1:** Clinical and Imaging characteristics of the study patients.

Variables	Training set						Validation set		
	IDH-wild	IDH-mutant	IDH-mutant (GAN)	*P* +	P*	P**	IDH-wild	IDH-mutant	*P*
No. of patients	80	38	38				33	11	
Median age (years)	60	47	–	–	< 0.001	< 0.001	58	42	= 0.003
**Enhancement category**				0.01	< 0.001	0.002			0.01
Rim enhancing	47	19	9				24	3	
Thick nodular	28	8	5				6	3	
Patch enhancing	5	11	24				3	5	
**Tumor location**				0.55	0.01	0.008			0.36
Frontal or insula	29	25	25				16	8	
Other	42	10	12				13	2	
Thalamus or brainstem	9	3	1				4	1	
**Necrosis**				0.35	0.001	< 0.001			0.007
Yes	72	23	19				30	6	
No	8	15	19				3	5	
**Surrounding high signal intensity**				0.39	0.001	< 0.001			< 0.001
Tumor dominant	35	29	32				8	11	
Edema dominant	45	9	6				25	0	
**Margin of non-enhancing lesion**				0.10	< 0.001	< 0.001			< 0.001
Clear	4	20	27				0	6	
Indistinct	76	18	11				33	5	

Data are expressed as the counts and median. *P* + indicates differences between real and synthetic IDH-mutant data. P* and P** indicate differences between real IDH-wild type and real IDH-mutant data and between real IDH-wild type and synthetic IDH-mutant data, respectively.

*IDH* isocitrate dehydrogenase.

### Evaluation of reality

**Figure 3:**
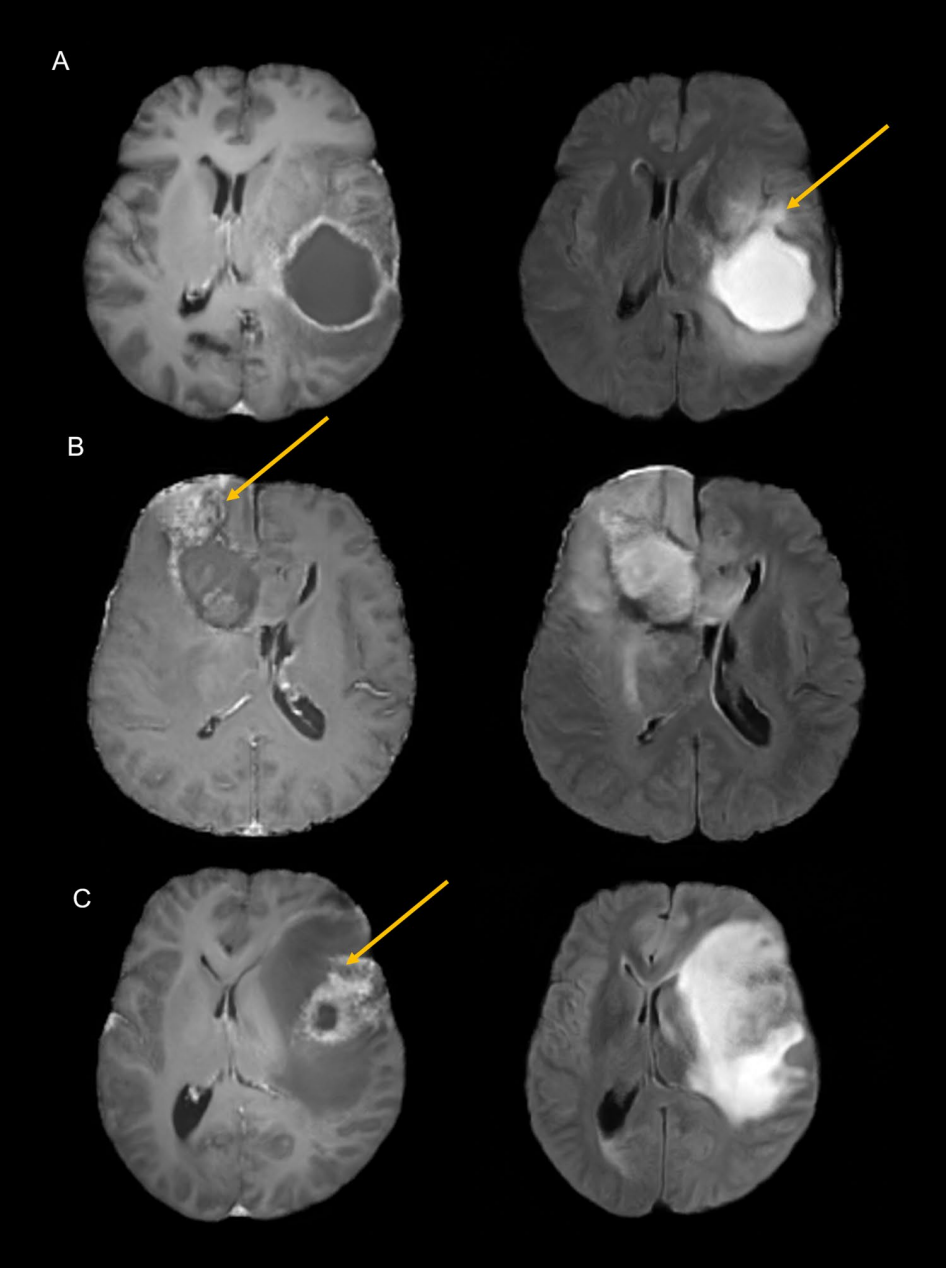
Representative synthetic images correctly determined to be synthetic by neuroradiologists. (**A**) Contrast-enhanced T1-weighted (CE-T1w) image similar to a real image, coupled with a FLAIR image showing an open rim of hypointensity, suggesting that the image was not real. (**B**) CE-T1w images showing nodular enhancement with a mesh-like artifact, suggesting that these images were not real. (**C**) CE-T1w images showing bizarre-shaped linear enhancement, suggesting that these images were not real.

Turing tests of the imaging data showed that the correct classification rates by readers 1 and 2 were 55% and 62%, respectively, showing that it was difficult to distinguish between real and synthetic data. Reader 1 misclassified 22 real images as synthetic, while misclassifying 23 synthetic images as real. Reader 2 misclassified 20 real images as synthetic, while misclassifying 18 synthetic images as real. Examples of synthetic data correctly classified as synthetic are shown in Fig. 3.

### Distribution of morphologic features

A comparison of imaging data of real and synthetic IDH-mutant glioblastomas showed no differences in tumor location (*x*^2^ test, *P* = 0.55), degree of necrosis (*P* = 0.35), and tissue (*P* = 0.39) and margins (*P* = 0.10) surrounding regions of high signal intensity The patch enhancing pattern was observed more frequently in the synthetic than in the real imaging data (*P* = 0.01). Frontal or insular location was significantly more frequent in both patient (*P* = 0.01) and synthetic (*P* = 0.008) data in the training set, but not in the validation set.

Compared with imaging of IDH-wild type glioblastoma, imaging of IDH-mutant type glioblastoma showed that rim enhancing pattern was less frequent in both patients (highest *P* = 0.01) and in the synthetic dataset (*P* = 0.002). Similarly, internal necrosis was significantly less frequent in IDH-mutant than in IDH-wild type in both patients (highest *P* = 0.001) and in the synthetic dataset (*P* < 0.001). By contrast, distinct margins surrounding areas of high intensity were significantly more common in IDH-mutant than in IDH-wild type in the patients (highest *P* = 0.001) and in the synthetic dataset (*P* < 0.002).

### Significant predictors of IDH mutation

The two readers showed moderate agreement regarding tumor location (κ = 0.67, *P* < 0.001), patterns of enhancement (κ = 0.67, *P* < 0.001), presence of necrosis (κ = 0.65, *P* < 0.001), and margins of non-enhancing lesions (κ = 0.56, *P* < 0.001).

Table 2 shows the results of univariable and multivariable logistic regression analyses. Multivariable analysis showed that, in both real and synthetic data, a more frontal or insular location (β = 1.34, *P* = 0.02 for real data; β = 1.52, *P* = 0.04 for synthetic data) and distinct margins of non-enhancing tumors (β = 2.68, *P* < 0.001 for real data; β = 3.88, *P* < 0.001 for synthetic data) were significant predictors of IDH mutation. Univariate analysis showed that absence of necrosis and presence of a patch enhancing pattern in both real and synthetic data were significant, whereas the multivariable model showed that the absence of necrosis was significant only for real data (β = 1.91, *P* = 0.02), and the presence of a patch enhancing patter was significant only for synthetic data (β = 3.46, *P* = 0.002).

**Table 2:** Univariable and multivariable binomial logistic regression analysis of factors predicting IDH mutation in the training dataset.

Variables	With real data (model 1) (n = 118)				With IDH-mutant synthetic data (model 2) (n = 118)				Augmented with real and synthetic data (model 3) (n = 156)			
	Univariate analysis		Multivariate analysis		Univariate analysis		Multivariate analysis		Univariate analysis		Multivariate analysis	
	Beta coefficient	*P*	Beta coefficient	*P*	Beta coefficient	*P*	Beta coefficient	*P*	Beta coefficient	*P*	Beta coefficient	*P*
**Tumor location**												
Other	Ref.		Ref.		Ref.		Ref.		Ref.		Ref.	
Frontal or insula	1.29	**0.004**	1.34 (1.24, 1.45)	**0.02**	1.10	0.009	1.52 (1.04, 2.35)	**0.04**	1.19	** < 0.001**	1.32 (1.24, 1.4)	**0.008**
Thalamus or brainstem	0.95	0.18	0.25 (0.09, 0.41)	0.77	2.05	0.06	3.33 (2.89, 3.77)	0.77	1.35	**0.03**	0.78 (0.64, 0.91)	0.36
Absence of necrosis	1.77	** < 0.001**	1.91 (1.77, 2.05)	**0.017**	2.19	< 0.001	− 0.06 (− 0.24, 0.12)	0.95	1.98	** < 0.001**	1.13 (− 0.52, 0.78)	0.08
**Enhancement category**												
Rim enhancing	Ref.		Ref.		Ref.				Ref.			
Thick nodular	− 0.35	0.47	− 1.50 (− 1.64, − 1.36)	0.05	− 0.07	0.90	0.25 (0.086,0.414)	0.78	− 0.25	0.54	− 0.58 (0.66,− 0.49)	0.27
Patch enhancing	1.69	**0.005**	0.035 (− 0.135, 0.205)	0.97	3.22	** < 0.001**	3.46 (3.26, 3.66)	**0.002**	2.46	** < 0.001**	1.97 (1.87, 2.07)	**0.002**
Edema− dominant surrounding high signal intensity	− 0.91	**0.04**	− 0.54 (− 0.646, − 0.434)	0.35	− 3.56	**0.001**	− 1.29 (− 1.5, − 1.08)	0.26	− 1.62	**0.001**	− 0.83 (− 0.91, − 0.75)	0.11
**Margin of non-enhancing lesions**												
Indistinct	Ref.		Ref.		Ref.		Ref.		Ref.		Ref.	
Distinct	2.94	** < 0.001**	2.68 (2.56, 2.8)	** < 0.001**	3.84	< 0.001	3.88 (3.04, 4.72)	** < 0.001**	3.47	< 0.001	2.96 (2.86, 3.06)	** < 0.001**

Data in parentheses are 95% confidence intervals.

### Diagnostic performance for IDH mutation

The results of diagnostic performance are shown in Table 3. The synthetic model (AUC 0.96; 95% CI 0.90–0.99) showed higher diagnostic performance than the real model (AUC 0.86; 95% CI 0.80–0.92) in the training set. In the validation set, the diagnostic performance was similar in both the real and synthetic model, with readers 1 and 2 showing AUCs of 0.71 (95% CI 0.54–0.89) and 0.77 (95% CI 0.56–0.98), respectively, for the real model, and AUCs of 0.75 (95% CI 0.52–0.98) and 0.77 (95% CI 0.56–0.98), respectively, for the synthetic model.

**Table 3:** Diagnostic performance of the models for prediction of IDH mutation.

	Model from real data (model 1)				Model from IDH-mutant synthetic data (model 2)				Combined with real and synthetic data (model 3)			
	AUC	95% CI	Sensitivity	Specificity	AUC	95% CI	Sensitivity	Specificity	AUC	95% CI	Sensitivity	Specificity
Training set	0.864	0.789, 0.920	57.9%	95.0%	0.958	0.904, 0.986	84.2%	92.5%	0.899	0.841, 0.942	72.37%	91.25%
**Validation set**												
Reader 1	0.713	0.535, 0.892	36.4%	100%	0.747	0.517, 0.978	63.6%	100%	0.747	0.517, 0.978	63.6%	100%
Reader 2	0.773	0.565, 0.981	62.7%	63.6%	0.773	0.565, 0.981	62.7%	63.6%	0.821	0.653, 0.989	63.6%	93.9%
**Combined with age information**												
Reader 1	0.871	0.722, 1.00	63.6%	100%	0.826	0.662, 0.991	63.6%	100%	0.826	0.662, 0.991	63.6%	100%
Reader 2	0.855	0.710, 1.00	78.8%	81.8%	0.861	0.720, 1.00	69.7%	90.9%	0.861	0.720, 1.00	69.7%	90.9%

*AUC* area under the receiver operating characteristics curve.

### Effect of data augmentation

Use of an augmented model, in which synthetic data were added to real data, showed the same predictors of IDH-mutant as the synthetic model, with a multivariable analysis showing that a more frontal or insular location (β = 1.32, *P* = 0.01), the presence of a patch enhancing pattern (β = 1.97, *P* = 0.002), and distinct margins of non-enhancing tumors (β = 2.96, *P* < 0.001) were statistically significant. In the training set, the augmented model had a diagnostic performance (AUC, 0.90; 95% CI, 0.84–0.94) slightly higher than that of the real model (AUC, 0.86) and slightly lower than that of the synthetic model (AUC, 0.96). In the validation set, the augmented model showed slightly higher diagnostic performance (AUC, 0.75 for reader 1 and 0.82 for reader 2) than the synthetic or real model. The augmented model had greater diagnostic accuracy (90.9% [40/44] and 93.2% [41/44] for readers 1 and 2, respectively) than the real model (84.1% [37/44] and 86.4% [38/44] for readers 1 and 2, respectively).

## Discussion

This study found that the morphologic characteristics exhibited by synthetic and real imaging data of IDH-mutant glioblastomas were generally similar, with the two datasets being similar in tumor location, margins, type of tissue surrounding areas of high signal intensity, and presence of necrosis, but not in contrast-enhancing patterns. Univariable analysis showed that the same morphologic characteristics, including tumor location, absence of necrosis, enhancement category, and margins and type of tissue surrounding non-enhanced regions, were predictive of IDH mutation in both the real and synthetic datasets. A multivariable diagnostic model derived from synthetic data showed higher predictive performance than a model derived from real data in the training set, with the two models having similar predictive performance in the independent validation set. Thus, the morphologic variations of GAN-based synthetic images of IDH-mutant glioblastomas was similar to that of actual images, suggesting that the former may serve as a realistic training set.

Models have shown the ability to distinguish between IDH-mutant and IDH-wild type gliomas with AUCs of 0.80–0.94 ^8–10,34^. Based on the prevalence of IDH-mutant glioblastomas, the sample size required for sufficient training for deep learning is up to 1200 patients. This number, however, is difficult to achieve in practice and requires data augmentation. Previous studies using GAN^20,35^ showed that augmentation with synthetic data improved the diagnostic performance of the model, but those studies were more limited in that performance was measured in the training set. The performance of the synthetic and augmented models in the present study was similar to or higher than the performance of the real-data only model in both the training and validation sets. In addition, age was an important predictor of IDH mutation status, suggesting that the synthetic data generated by GAN may be useful for extracting image-based morphologic features and could be combined with age as an additional predictor.

GAN may have the ability to learn the complete distribution of data when given “sufficiently large” deep networks, sample size, and computation time^36^. To utilize GAN to learn the characteristics of IDH-mutant glioblastoma, we first optimized the sample size for StyleGAN2, until GAN provided sufficiently realistic imaging data without artifacts. We then trained GAN with the images available for IDH-mutant high-grade astrocytomas to generate synthetic images and transfer them to IDH-mutant glioblastomas. This provided important evidence about training on a rare disease, generating certain types of images, such that style transfer could be useful for a pre-trained network to improve image quality (image reality). Subsequently, a specific outcome, such as a certain molecular subtype or diagnosis, would be appropriate in a latent space. The synthetic images created in the present study showed similar but not identical morphology to the training dataset, providing a smooth transition in the latent space^37^ with the GAN network.

Two-channel GAN was able to simultaneously generate contrast-enhanced T1-weighted (CE-T1w) and FLAIR images. This is important for GAN-based synthetic images because both images are necessary to characterize IDH mutations and may be useful for data augmentation in deep learning. Two-channel GAN can fully determine the morphologic characteristics of conventional imaging data predictive of IDH mutation, including focal patch enhancement within areas of high signal intensity on FLAIR^8,9^, and distinct margins of non-enhancing lesions^8,10^ determined by high signal intensity on FLAIR without contrast enhancement. Univariate analysis of all three models, the real, synthetic, and augmented models, yielded the same predictive factors, indicating that the distribution of morphologic variations was similar for real and synthetic data, and suggesting that the use of synthetic data for diagnostic training was feasible.

This study had several limitations. First, synthetic data were generated from IDH-mutant high-grade astrocytomas, not solely from glioblastomas, in which patchy enhancing patterns were more frequent. High-grade astrocytomas were included in GAN training because the IDH-mutant glioblastomas available for GAN training was small. Second, although this study included qualitative imaging features from structural MRI with high reproducibility in several studies^8,9,38^, physiologic imaging biomarkers can be helpful in differentiating IDH-mutant glioblastoma, demonstrating less aggressive imaging features with higher ADC values and less hyperperfusion on CBV than IDH-wild type glioblastoma^38–40^. Also, characteristics imaging phenotype of T2/FLAIR mismatch sign^41,42^ will be a future topic of image generation. The application of GAN for multi-contrast MRI generation has been previously proposed^16,17^, and generation of ADC and CBV are future goals to pursue, while adding quantitative analysis will improve the accuracy of molecular prediction. Third, sampling from GAN networks was random. The development of diagnostic models may depend on the sampling method. A more objective analysis requires the methodologic construction and testing of multiple diagnostic models, as well as their statistical improvement in the future.

In conclusion, the GAN-based synthetic images yielded morphologically variable, realistic but unseen IDH-mutant glioblastomas, and they were useful as realistic training sets to improve diagnostic performance. Our results provided evidence that synthetic IDH-mutant glioblastomas improved the visual diagnosis of tumors with IDH mutations and demonstrated the potential to improve noninvasive identification of IDH-mutant tumors, thus overcoming the small sample size inherent in imaging-based genomic and molecular prediction.

## Supplementary Information

Supplementary Figures.

## Data Availability

The datasets generated during and/or analyzed during the current study are available from the corresponding author on reasonable request.
