# Supplementary material for: Generative adversarial network for glioblastoma ensures morphologic variations and improves diagnostic model for isocitrate dehydrogenase mutant type
Source: Sci Rep. 2021 May 10;11:9912. doi: 10.1038/s41598-021-89477-w (PMC8110557; doi:10.1038/s41598-021-89477-w)
Supplement: Supplementary file 1 — Supplementary Figures. [file 41598_2021_89477_MOESM1_ESM.docx]

**Supplementary Materials for**

**Generative Adversarial Network for Glioblastoma Ensures Morphologic Variations and Improves Diagnostic Model for Isocitrate Dehydrogenase Mutant Type**

Ji Eun Park^1*^, Dain Eun^2*^, Ho Sung Kim^1^, Da Hyun Lee^1^, Ryoung Woo Jang^2^, and Namkug Kim^1,2^

^1^ Department of Radiology and Research Institute of Radiology, University of Ulsan College of Medicine, Asan Medical Center, Seoul 05505, Korea

^2^ Department of Convergence Medicine, University of Ulsan College of Medicine, Asan Medical Center, Seoul 05505, Korea

*These authors contributed equally to the manuscript.

**Corresponding author**: Ho Sung Kim, M.D., Ph.D.

Department of Radiology and Research Institute of Radiology, University of Ulsan College of Medicine, Asan Medical Center, 43 Olympic-ro 88, Songpa-Gu, Seoul 05505, Korea

Phone: 82-2-3010-5682

E-mail address: radhskim@gmail.com

**Supplementary Figure and Figure Legends**

**
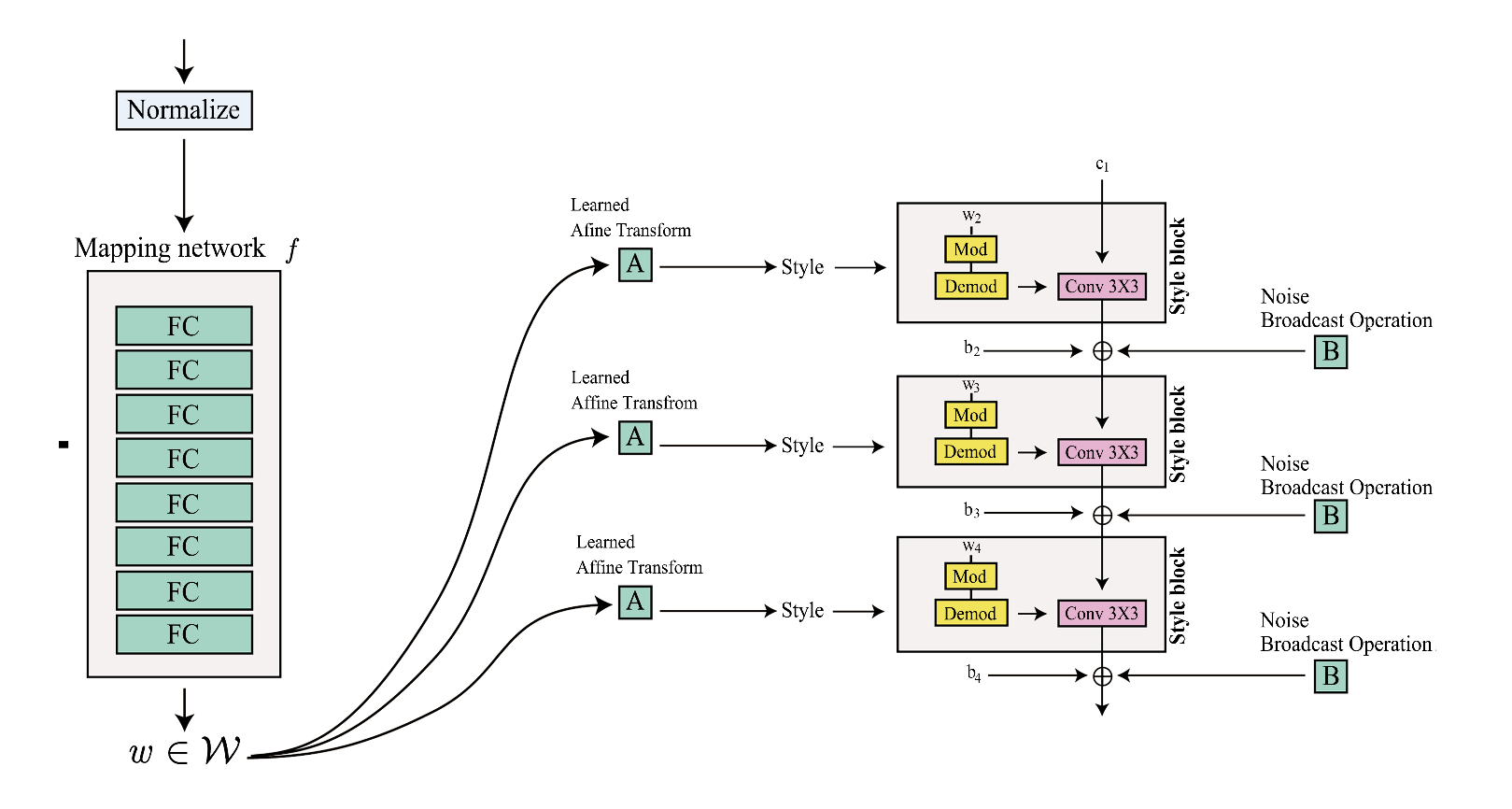
**

**Supplementary Figure 1.** The network architecture of StyleGAN2. The mapping network $f: \mathcal{Z\to W}$ transforms latent $z\mathcal{\in Z}$ from uniform distribution to the intermediate latent vector $w\mathcal{\in W}$. Styles are generated from learned transformation of $w$. Each feature map was processed with modulation and demodulation operations. Bias and noise operations were added outside the styleblock. "FC" indicates fully connected layer, "A" indicates learned affine transform, and "B" indicates noise broadcast operation. w, b, and c indicate learned weight, bias, and constant, respectively.

**
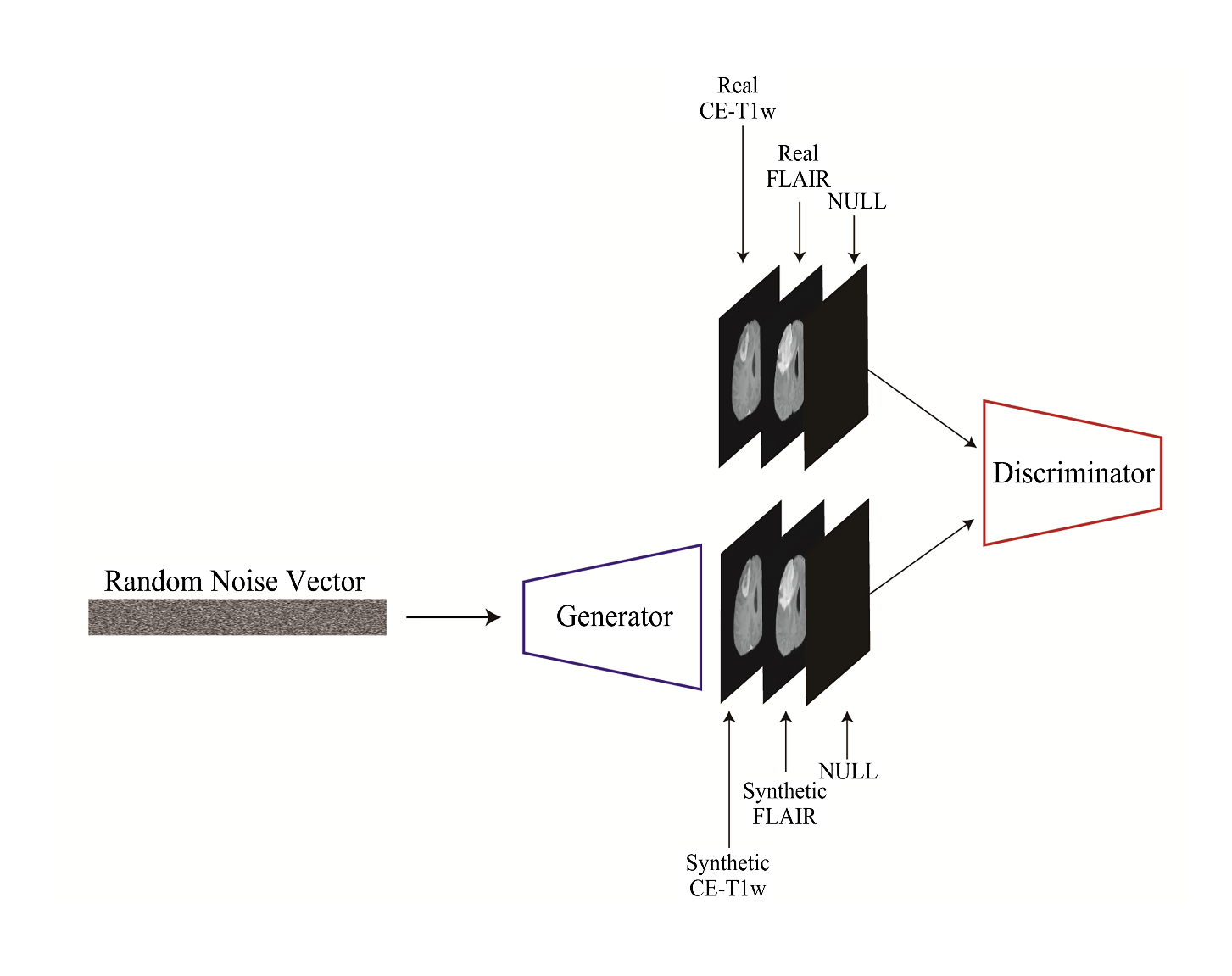
**

**Supplementary Figure 2.** The network architecture of StyleGAN2 using two-channel contrast-enhanced T1-weighted imaging (CE-T1w) and FLAIR.


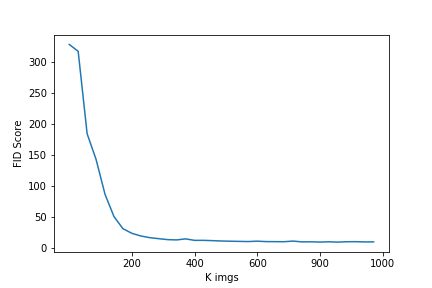


**Supplementary Figure 3.** The Fréchet inception distance (FID) score shows a smooth decrease from 327 points to below 9.5 points as the network was iteratively trained, with stabilized with 500 k images.

**
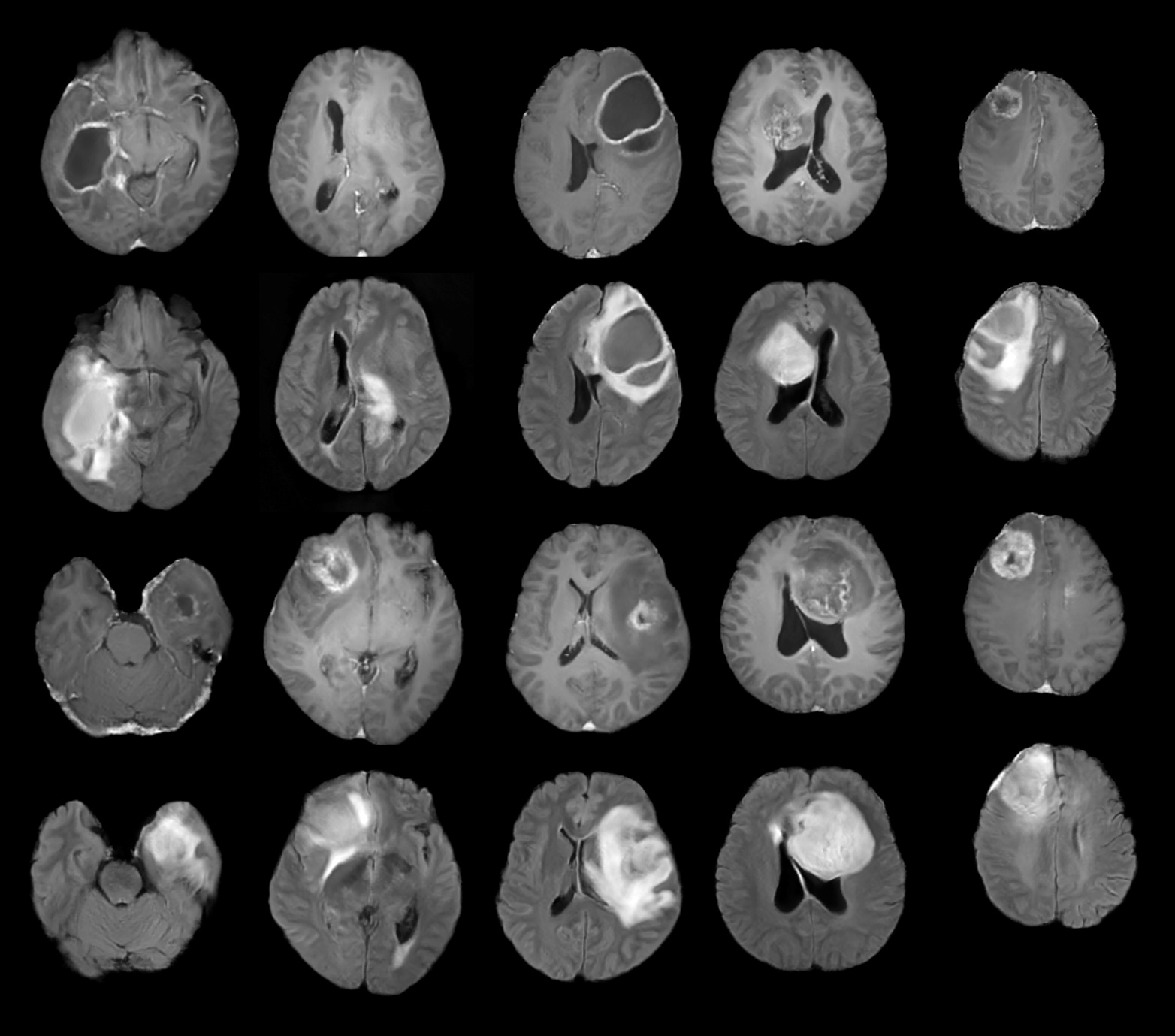
**

**Supplementary Figure 4.** Generated synthetic imaging data obtained from the training set of IDH-mutant high-grade astrocytomas. The synthetic image showed realistic image quality, distinct margins of non-enhancing lesions, and patchy enhancing patterns, representing IDH-mutant molecular status.
